# Cell type-specific differences in herpes simplex virus type 1 infection and dependency on ICP27

**DOI:** 10.1128/jvi.00052-26

**Published:** 2026-02-26

**Authors:** Sabrina L. Rutan, Jill A. Dembowski

**Affiliations:** 1 Department of Biological Sciences, Duquesne University 189492, Pittsburgh, Pennsylvania, USA; University of Virginia, Charlottesville, Virginia, USA

**Keywords:** herpes simplex virus type 1 (HSV-1), infected cell protein 27 (ICP27), cell type specificity, infectious cycle, lytic infection, N/TERT-2G, MRC-5, Vero, HeLa, HFF

## Abstract

While many cell types are permissive to herpes simplex virus type 1 (HSV-1) infection, the efficiency and outcome of infection can vary significantly depending on the cell type infected. In addition, viral mutants may replicate more favorably in one cell type compared to another. Here, we sought to identify key differences in the infectious cycle of HSV-1 and infection in the absence of infected cell protein 27 (ICP27) in several cell types used for HSV-1 research. These include African green monkey kidney epithelial (Vero), human lung fibroblast (MRC-5), human foreskin fibroblast (HFF), human diploid keratinocyte (N/TERT-2G), and human cervical cancer epithelial (HeLa) cells. ICP27 is an essential viral immediate early gene product that promotes efficient processing and transport of viral mRNAs. Based on previous studies, replication of an ICP27 mutant virus is cell type-dependent. Here, we demonstrate that the kinetics of wild-type infection, including the timing of the onset of viral DNA replication, mRNA and protein expression, and infectious virus output, are also cell type-dependent. We also found that while infection with an ICP27 deletion mutant leads to a decrease in viral mRNA and protein expression in all cell types tested, some cell types are more reliant on ICP27 for viral DNA replication. Together, this study highlights cell type-specific differences in HSV-1 infection and underscores the importance of cellular context in determining the efficiency of infection and reliance on ICP27.

**IMPORTANCE:**

HSV-1 research has been completed in multiple cell types, which have inherently different characteristics. This study analyzes the nuclear stages of HSV-1 infection in multiple cell types commonly used in HSV-1 research. We illustrate the cell type specificity of HSV-1 infection with respect to viral DNA replication, mRNA expression, protein expression, infectious virus output, and the effect a viral mutation has on these processes. These data reveal significant differences in infection depending on the cell type infected and could serve as a resource for future HSV-1 research. This study also underscores potential limitations when comparing data collected across different cell types, as results may be cell type-specific.

## INTRODUCTION

Herpes simplex virus type 1 (HSV-1) infects 64% of the human population but can affect up to 90% of the adult population depending on the geographic location (1). HSV-1 initially infects epithelial cells and establishes latency in the neurons of the sensory ganglia. When stressed, the virus can reactivate, resulting in recurrent sores at the initial site of infection. Infections can range from blisters on the skin to more severe cases that can result in herpes keratitis or encephalitis (2–4). Herpes keratitis is an infection of the eye and is the leading cause of infectious blindness in the developed world (5, 6). There are no preventative HSV-1 vaccines, and there is no cure. HSV-1 can generally be managed with antivirals that reduce frequency and intensity but do not eliminate the latent pool of virus (7). HSV-1 antivirals primarily target viral DNA replication (8). However, viral mutations can lead to antiviral resistance (9).

The HSV-1 genome is approximately 152 kilobase pairs and codes for over 80 genes that are temporally transcribed by host RNA polymerase II (RNAPII) (10–12). Viral genes are classified as immediate early (IE), early (E), leaky late (LL), or true late (L) genes. IE genes are expressed once the viral genome enters the nucleus and include transcription factors and regulatory proteins. IE genes stimulate E gene expression, which includes the viral DNA replication machinery. Viral DNA replication, in turn, stimulates LL and L gene expression (13). LL genes are expressed at low levels before viral DNA replication, and their expression is amplified post-replication. L genes are expressed after the replication of viral DNA and mostly code for structural and packaging proteins.

Several different cell types are used to study productive HSV-1 infection. However, the rate and efficiency of the infectious cycle can vary between cell types. Epithelial cells, including keratinocytes, are physiologically relevant cells, since they are the primary site of initial HSV-1 infection and where productive infection occurs. Common cell types used in HSV-1 research include Vero (African green monkey kidney epithelial), HeLa (human cervical cancer epithelial), and various fibroblast cells such as MRC-5 (human lung fibroblast) or HFF (human foreskin fibroblast) cells. In addition, more recent studies have used N/TERT-2G cells, a telomerase-immortalized human diploid keratinocyte cell line, due to their relevance to natural infection (14–17). N/TERT-2G cells could provide a reproducible and scalable alternative for HSV-1 research.

All cells used in research serve the purpose of modeling various aspects of infection. In this study, we set out to define how cell type can influence the outcome of HSV-1 infection. We specifically compared the infectious cycle during infection of Vero, HeLa, HFF, MRC-5, and N/TERT-2G cells. Previous research has shown that infected N/TERT-2G cells express viral proteins approximately 2 h before Vero cells and produce progeny virus approximately 4 h before Vero cells (16); therefore, there could be specific differences in the infectious cycle depending on the cell type infected. In addition, cell type-specific differences in the infectious cycle can be amplified by viral mutant phenotypes.

Among other factors, cell type specificity is directly related to mutations in infected cell protein 27 (ICP27), an essential IE gene product (18). ICP27 is the only IE gene with homologs in all human herpesviruses (19, 20). This protein regulates viral gene expression through several mechanisms, including splicing, 3´ end processing, and mRNA export (21, 22). Cellular RNAPII is hijacked by HSV-1, and ICP27 regulates transcription through association with the C-terminal domain of RNAPII. This association leads to the inhibition of host genes through blocking transcription termination and disrupting host splicing (22, 23). ICP27 aids in viral mRNA export through the leucine-rich nuclear export signal (NES) on the N-terminus of the protein (21, 24–26). ICP27 physically interacts with intronless viral mRNAs, ensuring preferential nuclear export of unspliced viral mRNAs over cellular mRNAs (23). A recent study found that DNA replication of an ICP27 NES mutant virus is cell type-dependent (18). Previously, the HSV-1 ICP27 protein has been characterized as a multifunctional IE protein. However, the multiple functions of this protein have been characterized in various cell types, including Vero, HeLa, and Hep-2 cells, potentially complicating the comparison of data between different experiments (22, 26, 27).

We set out to characterize the HSV-1 infectious cycle in cell types used across the HSV-1 research field. We found that infection of the HSV-1 strain KOS is different among cell types in terms of the dynamics of viral DNA replication, gene and protein expression, and viral output. We also found that infection with an ICP27 deletion mutant proceeds differently depending on the cell type infected. Therefore, ICP27 may have cell type-specific functions.

## RESULTS

### Cell type-specific kinetics of HSV-1 DNA replication

**Fig 1:**
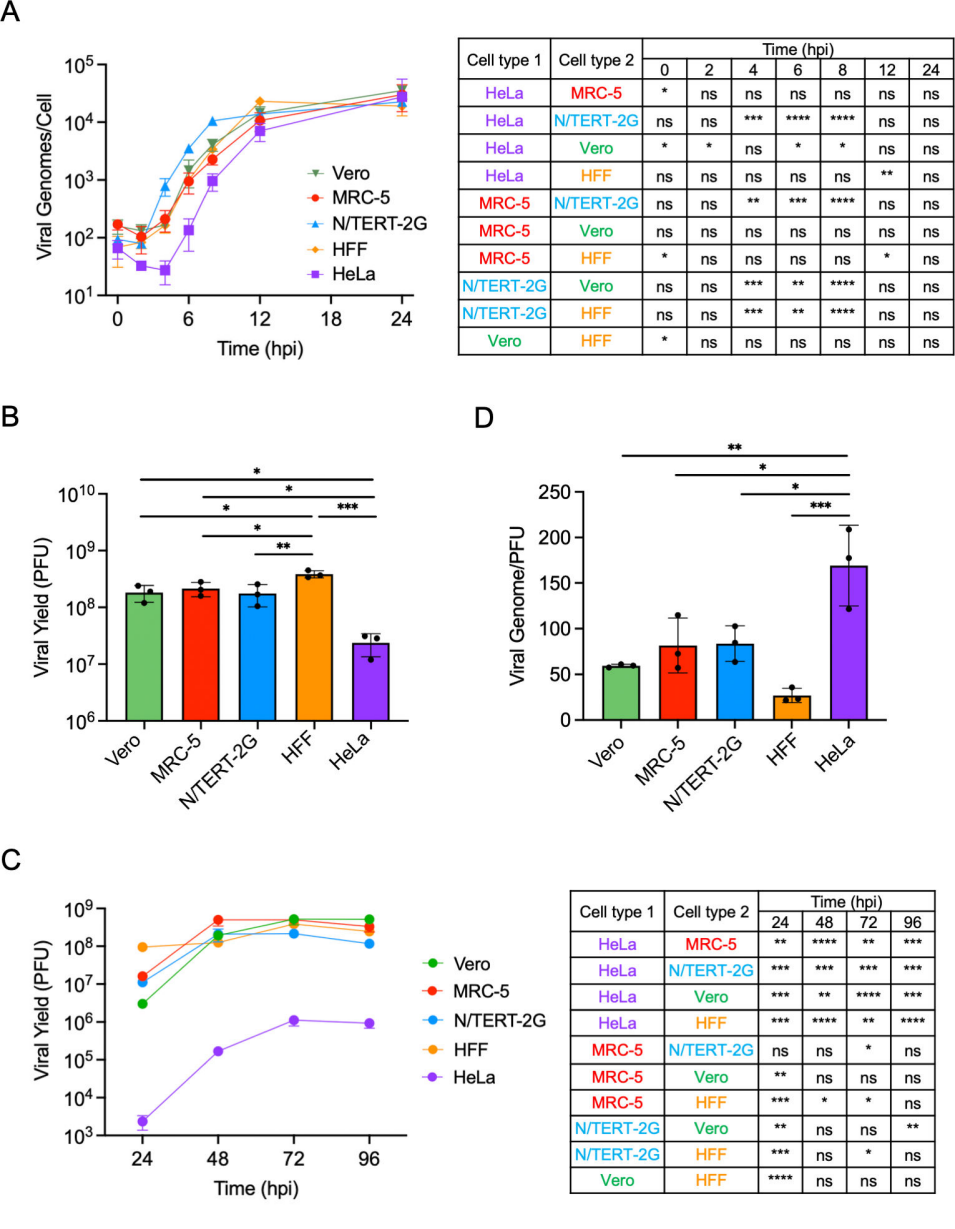
Characterization of HSV-1 DNA replication and infectious virus output in several cell types. (**A**) Comparison of viral DNA replication kinetics between cell types. Vero, MRC-5, N/TERT-2G, HFF, or HeLa cells were infected with strain KOS at an MOI of 10 PFU/cell. Total DNA was collected every 2 h for 12 h and at 24 hpi. The quantities of cellular and viral genomes were calculated via qPCR relative to standard curves generated from known amounts of purified human or viral DNA. Viral genomes per cell were then determined. All values represent the mean with standard deviation (*n* = 3). A one-way ANOVA with Tukey’s multiple comparisons test was completed to compare the differences between each cell type at each time point (ns not significant, *P < 0.05, **P < 0.01, ***P < 0.001, ****P < 0.0001). (**B**) Comparison of viral yield between cell types after high MOI infection. Cells were infected with KOS at an MOI of 10 PFU/cell, virus was collected at 24 hpi, and viral yield was determined by plaque assay in Vero cells. All values represent the mean with standard deviation (*n* = 3). A one-way ANOVA with a Tukey’s multiple comparisons test was completed to compare the differences between each experimental group (*P < 0.05, **P < 0.01, ***P < 0.001, ****P < 0.0001). Differences that were not significant are not indicated on the graph. (**C**) Comparison of viral yield after low MOI infection. Cells were infected with KOS at an MOI of 0.01 PFU/cell. Virus was collected at 24, 48, 72, and 96 hpi, and viral yield was determined by plaque assay in Vero cells. A Brown-Forsythe and Welch ANOVA test with a Dunnett’s T3 multiple comparisons test was completed on log10-transformed data to compare viral yield at each time point in each cell type (*P < 0.05, **P < 0.01, ***P < 0.001, ****P < 0.0001). Differences that were not significant are not indicated on the graph. (**D**) Comparison of defective virus particles between cell types. Cells were infected with KOS at an MOI of 10 PFU/cell and supernatant virus was collected at 24 hpi. The number of encapsidated viral genomes was determined by a proteinase K digestion, followed by qPCR.Infectious virus was determined by plaque assay in Vero cells. All values represent the mean with standard deviation (*n* = 3). A one-way ANOVA with a Tukey’s multiple comparisons test was completed to compare the differences between each experimental group. (*P < 0.05, **P < 0.01, ***P < 0.001, ****P < 0.0001). Differences that were not significant are not indicated on the graph.

To begin to characterize cell type-specific differences in the HSV-1 infectious cycle, we compared the kinetics of viral DNA replication between cell types. Vero, MRC-5, N/TERT-2G, HFF, or HeLa cells were infected with HSV-1 strain KOS at a multiplicity of infection (MOI) of 10 plaque-forming units (PFU)/cell. Infected cells were harvested, and DNA was collected at 2-h intervals before completing quantitative real-time PCR (qPCR) to calculate the number of viral genomes per cell (Fig. 1A). Although viral entry appears to be similar between all cell types, the number of infecting viral genomes decreased shortly after infection in HeLa cells, and viral DNA replication was delayed. Replication kinetics were most similar between Vero, MRC-5, and HFF cells, and replication appeared to begin earlier and at a faster rate in N/TERT-2G cells. Although the timing of the onset of viral DNA replication and individual replication rates varied between cell types, all cell types had a similar maximum capacity to replicate viral DNA, reaching 20,000–30,000 viral genomes/cell by 24 h post-infection (hpi).

### HSV-1 produces less progeny virus in HeLa cells than in other cell types

We next determined the quantity of infectious viral progeny produced by each cell type when infected with the HSV-1 strain KOS. Vero, MRC-5, N/TERT-2G, HFF, or HeLa cells were infected with KOS at an MOI of 10 PFU/cell. After 24 h, virus was collected, and yield was determined by plaque assays in Vero cells (Fig. 1B). Vero, MRC-5, and N/TERT-2G cells all produced ~2 × 10^8^ PFU when 2.5 × 10^5^ cells were infected with KOS, whereas HFF cells produced ~3.8 × 10^8^ PFU and HeLa cells produced ~2.4 × 10^7^ PFU. This resulted in the production of ~800 PFU/cell in Vero, MRC-5, and N/TERT-2G cells, ~1,500 PFU/cell in HFF cells, and only ~100 PFU/cell in HeLa cells. Although all cell types produced similar quantities of viral genomes/cell at 24 hpi (Fig. 1A), HeLa cells were less efficient at producing infectious virus (Fig. 1B).

To confirm the observation that the capacity to produce infectious virus is different depending on the cell type infected, viral yield was determined during multistep infection in all cell types after infection at a low MOI. Vero, MRC-5, N/TERT-2G, HFF, or HeLa cells were infected with KOS at an MOI of 0.01 PFU/cell. Virus was collected at 24, 48, 72, or 96 hpi, and viral yield was determined by plaque assay in Vero cells (Fig. 1C). Like single-step, high MOI infection, HFF cells produced larger quantities of infectious viral progeny at 24 hpi, and HeLa cells produced less infectious virus throughout the time course. Vero, MRC-5, N/TERT-2G, and HFF cells produced, on average, ~1,000 PFU/cell by 96 hpi, whereas HeLa cells produced only 4 PFU/cell. This observation is consistent with HeLa cells producing significantly less infectious virus during single-step infection and further suggests decreased infectivity of progeny virus in subsequent rounds of infection.

### HeLa cells produce more defective virus than other cell types

Although HeLa cells produced the same number of viral genomes as the other cells during single-step infection, they produced significantly less infectious virus, which was amplified during multistep infection. This suggests that HeLa cells produce more defective virus than other cell types. To address this, we measured the ratio of packaged viral genomes to infectious virus collected from the cell-free supernatants of infected cells (Fig. 1D). Vero, MRC-5, N/TERT-2G, HFF, or HeLa cells were infected at an MOI of 10 PFU/cell. After 24 h, the supernatant was collected from infected cells. An aliquot of the supernatant was subjected to proteinase K digestion, followed by DNA isolation, and the number of viral genomes was quantified by qPCR. The remainder of the supernatant was used to determine the viral yield by plaque assay in Vero cells. We found that, on average, Vero cells produced 60 defective particles per PFU; MRC-5 and N/TERT-2G cells produced 82; HFF cells produced 27; and HeLa cells produced 170. Taken together, these data support the hypothesis that HeLa cells produce more defective virus than the other cell types tested and further indicate that HFF cells are the most efficient at producing infectious virus.

### Comparison of the kinetics of viral protein expression between cell types

**Fig 2:**
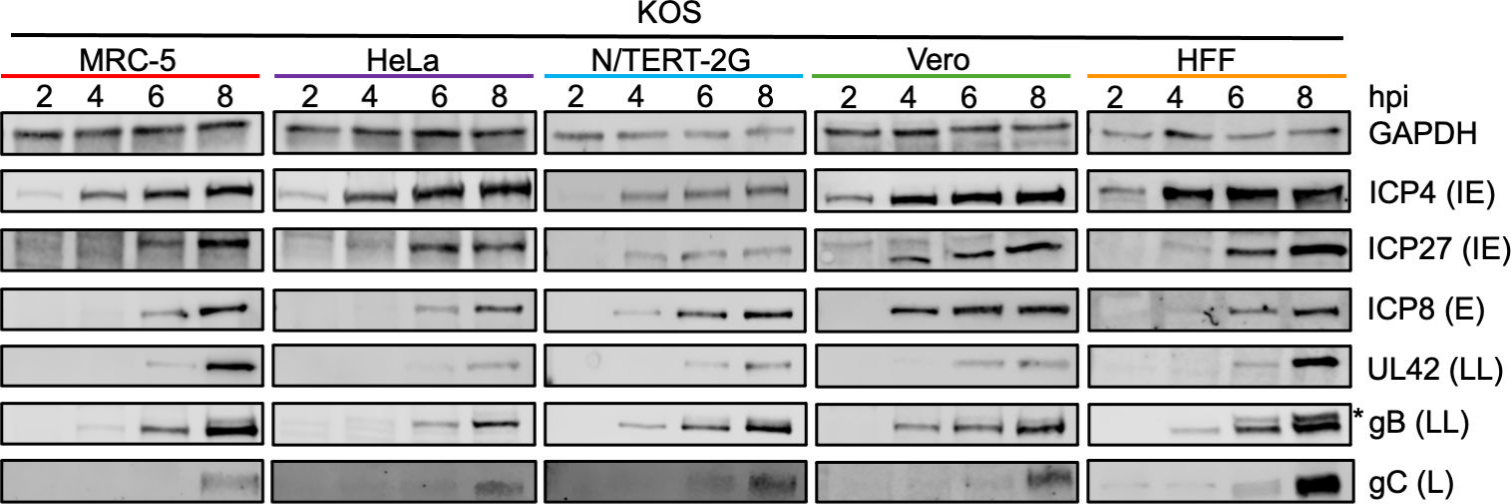
Comparison of HSV-1 strain KOS protein expression between cell types. MRC-5, HeLa, N/TERT-2G, Vero, or HFF cells were infected with KOS at an MOI of 10 PFU/cell. Total protein was collected at 2, 4, 6, or 8 hpi. Blots were incubated with antibodies indicated on the right of the images. * indicates an additional, potentially nonspecific band recognized by the gB antibody.

We next examined the timing of viral protein expression. MRC-5, HeLa, N/TERT-2G, Vero, or HFF cells were infected with KOS at an MOI of 10 PFU/cell; whole cell lysates were collected at 2, 4, 6, and 8 hpi; and representative IE, E, LL, and L viral proteins were detected by western blotting (Fig. 2). Cellular glyceraldehyde 3-phosphate dehydrogenase (GAPDH) is shown as a loading control and is only used to show consistent loading between time points within individual cell types because GAPDH levels differ between cell types (28).

We examined the expression kinetics of representative viral proteins from each HSV-1 gene class (11–13, 29). In general, for all cell types, the temporal expression of viral proteins was as expected. ICP4, the major viral transcription factor, was detected in all cell types by 2 hpi except for N/TERT-2G cells, for which it was detected by 4 hpi. In general, ICP4 levels increased and then plateaued by 6 hpi. ICP27 expression was consistently delayed compared to ICP4 expression and was detected between 4 and 6 hpi. Interestingly, the temporal expression of ICP27 was quite variable between cell types. ICP8, a representative E gene product that binds to single-stranded viral DNA during replication and recombination, was detected in MRC-5, HeLa, and HFF cells at 6 hpi, and the protein levels increased over time. After infection in N/TERT-2G cells, ICP8 was detected by 4 hpi with increasing protein levels over time. Although Vero cells had detectable levels of ICP8 at 4 hpi, ICP8 levels did not increase from 6 to 8 hpi. LL and L protein expression kinetics were similar between the five cell types, with different timing of expression between individual proteins. UL42 is an accessory subunit of the viral DNA polymerase that acts to increase the processivity of viral DNA replication (30). gB and gC are glycoproteins used for HSV-1 entry into cells (31). In all cell types, LL gene products UL42 and gB followed the same trends, where gB was detected 2 h before UL42, and the quantity of these proteins increased over time. LL protein expression was similar between MRC-5, N/TERT-2G, HFF, and Vero cells, while LL protein expression appeared to be delayed in HeLa cells. For all cell types tested, gC, a L gene product, was detected at 8 hpi. However, gC was detected at a low level at 6 hpi in N/TERT-2G and HFF cells. Taken together, although the temporal expression of viral proteins is consistent between cell types, with LL and L gene products expressed after the onset of viral DNA replication, each cell type is unique in the exact kinetics of viral protein expression. These differences may contribute to the differences observed in viral DNA replication (Fig. 1A) and infectious virus production (Fig. 1B through D) between the cell types tested.

### Viral mRNAs are expressed in a cell type-specific manner

**Fig 3:**
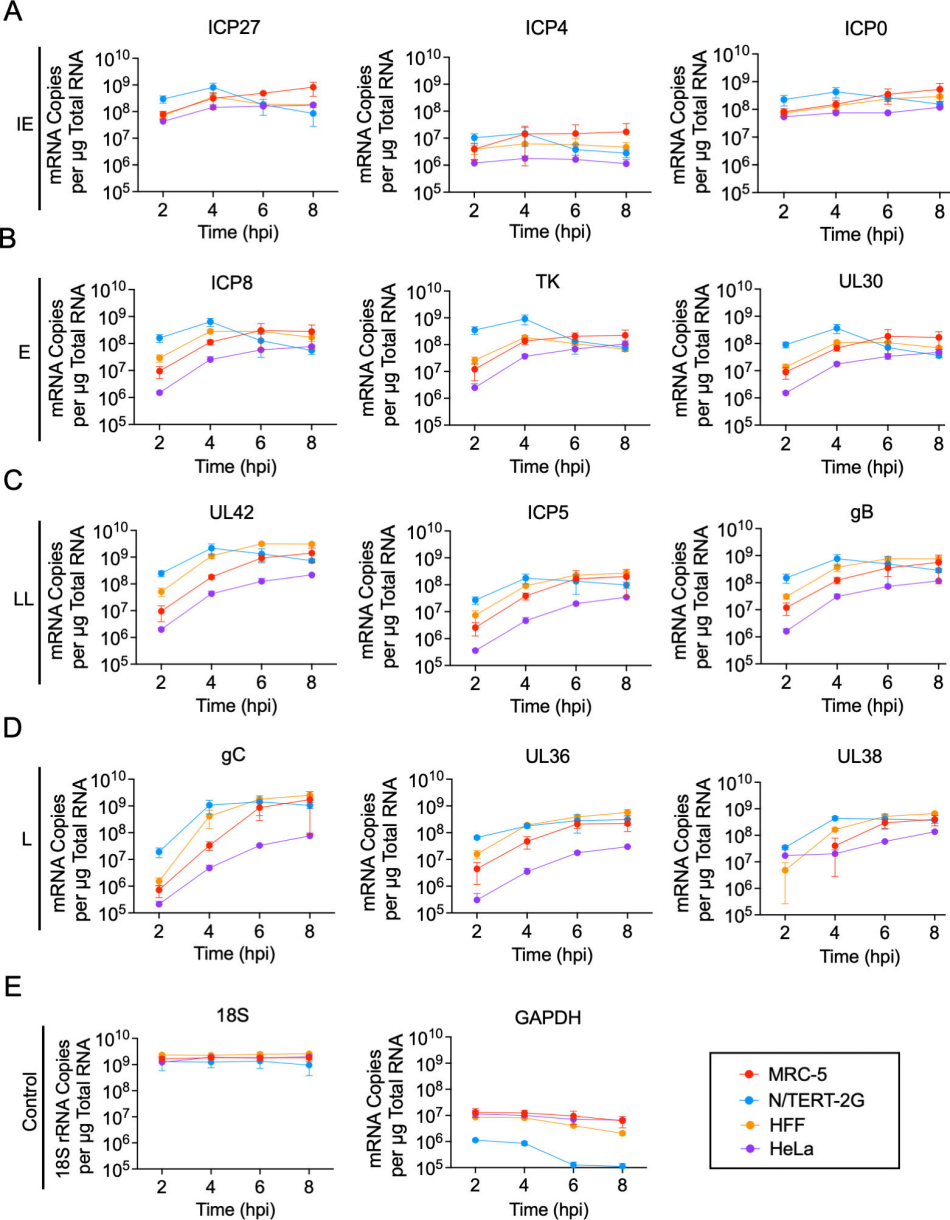
Viral gene expression kinetics vary between cell types during strain KOS infection. MRC-5, N/TERT-2G, HFF, or HeLa cells were infected with KOS at an MOI of 10 PFU/cell. Total RNA was isolated every 2 h for 8 h. After isolation, RNA was reverse-transcribed using oligo dT primers, and mRNAs were quantified by qPCR as follows: (**A**) IE genes, (**B**) E genes, (**C**) LL genes, and (**D**) L genes. (**E**) 18S rRNA was reverse-transcribed using a gene-specific primer and quantified by qPCR. Viral mRNA copies per microgram total RNA were determined via standard curve for each gene. All values represent the mean with standard deviation (*n* = 3). A one-way ANOVA with a Tukey’s multiple comparisons test was completed to compare the differences between each cell type (ns not significant, *P < 0.05, **P < 0.01, ***P < 0.001, ****P < 0.0001) (Fig. S1 and S2).

To investigate how cell type affects viral gene expression, we quantified the expression of several representative viral transcripts for each gene class. MRC-5, N/TERT-2G, HFF, or HeLa cells were infected at an MOI of 10 PFU/cell. Total RNA was isolated at 2, 4, 6, or 8 hpi, followed by DNase treatment, reverse transcription, and qPCR to quantify mRNA copies of each gene per μg total RNA (Fig. 3A through E). 18S ribosomal RNA (rRNA) was used as a control to compare relative mRNA levels between cell types. Although GAPDH was used as a loading control to compare protein expression over time in each cell type (Fig. 2), GAPDH levels can vary depending on the cell type infected. In contrast, 18S rRNA is highly expressed and stable across different cell types and experimental conditions (32). As shown in Fig. 3E and 5E, 18S rRNA levels are similar regardless of cell type or infecting virus. For statistical analysis comparing mRNA levels between cell types at each individual time point, see Fig. S1 and S2.

In general, viral mRNA levels were the highest in N/TERT-2G cells compared to the other cell types at early times after infection (Fig. 3). However, by 8 hpi, mRNA levels were similar in HFF, MRC-5, and N/TERT-2G cells for all HSV-1 gene classes. For N/TERT-2G cells, viral IE and E genes were expressed until 4 hpi and then steadily decreased (Fig. 3A and B). In HFF, MRC-5, and HeLa cells, IE and E gene expression mirror each other, where IE and E mRNA levels increase from 2 to 4 hpi and then plateau from 4 to 8 hpi. In N/TERT-2G cells, LL gene expression peaked at 4 hpi and then decreased as the infection proceeded, whereas L gene expression plateaued from 4 to 6 hpi (Fig. 3C and D). In HFF, MRC-5, and HeLa cells, LL and L gene expression increased steadily throughout infection, with more LL and L transcripts detected in all other cell types compared to HeLa cells. mRNA expression patterns are consistent with protein expression data in each cell type (Fig. 2). Consistently, fewer E, LL, and L viral transcripts were detected in HeLa cells compared to other cell types.

### Viral DNA replication in the absence of ICP27 is cell type-dependent

**Fig 4:**
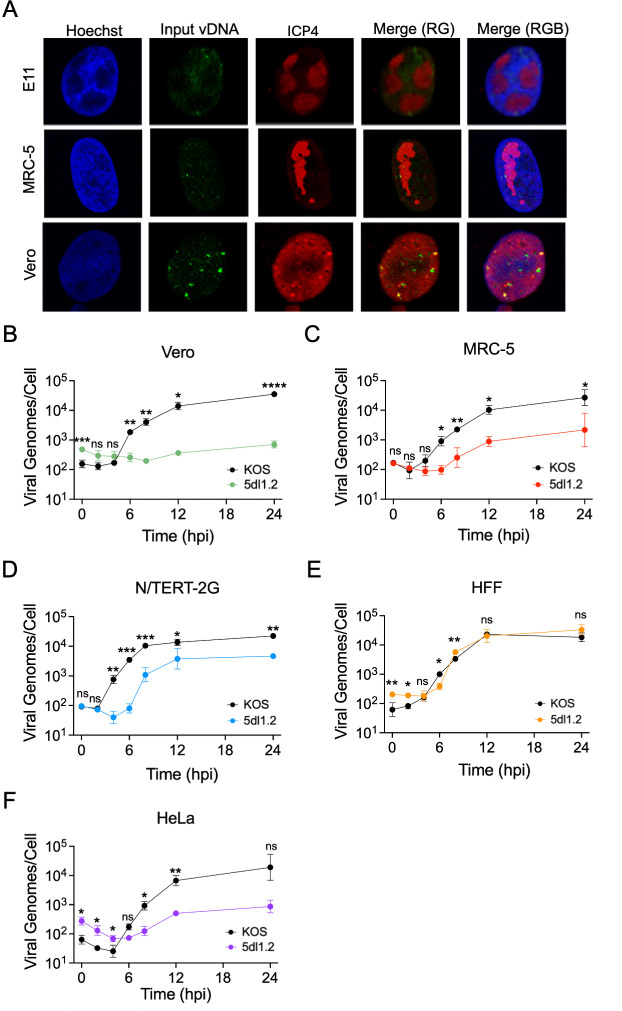
The dependence of viral DNA replication on ICP27 is cell type-specific. (**A**) Imaging of viral replication compartments. Immunofluorescence images of E11, MRC-5, and Vero cells infected with strain 5dl1.2 containing EdC-labeled viral DNA at an MOI of 10 PFU/cell. Cells were fixed at 6 hpi; cellular chromatin was stained with Hoechst (blue); viral DNA binding protein ICP4 was labeled by indirect immunofluorescence (red); and infecting viral DNA was visualized by click chemistry (green). ICP4 is the viral dsDNA binding protein that is used to label all viral DNA in the cell and marks incoming viral genomes and viral replication compartments. (**B–F**) Analysis of viral DNA replication. Vero (**B**), MRC-5 (**C**), N/TERT-2G (**D**), HFF (**E**), or HeLa (**F**) cells were infected with either strain KOS or 5dl1.2 at an MOI of 10 PFU/cell. Total DNA was collected every 2 h for 12 h and at 24 hpi. The number of cellular and viral genomes was quantified via qPCR relative to purified human or viral DNA standard curves. The number of viral genomes per cell was then calculated. All values represent the mean with standard deviation (*n* = 3). An unpaired two-tailed Student’s *t*-test was completed at each time point to compare the number of KOS and 5dl1.2 viral genomes/cell (ns not significant, *P < 0.05, **P < 0.01, ***P < 0.001, ****P < 0.0001).

In addition, we investigated cell type-specific differences in infection in the absence of ICP27, focusing first on viral DNA replication. The first evidence that there are cell type-specific differences in viral DNA replication was immunofluorescence imaging of viral replication compartments in MRC-5 and Vero cells. MRC-5 and Vero cells were plated on coverslips and infected with an EdC-labeled ICP27-deletion HSV-1 strain (5dl1.2) at an MOI of 10 PFU/cell. At 6 hpi, the cells were fixed, and EdC-labeled infecting viral genomes were detected by click chemistry to tag EdC-labeled DNA with a fluorophore (Fig. 4A). In addition, incoming viral DNA and viral replication compartments were labeled by indirect immunofluorescence to detect ICP4 because ICP4 binds to all double-stranded viral DNA. We found that viral replication compartments form in MRC-5 cells when ICP27 is absent. However, in Vero cells, incoming viral genomes form small punctate structures throughout the cell that do not become larger by 6 hpi. As a control, ICP27 complementing Vero cells (E11) were also infected with EdC-labeled 5dl1.2, and viral replication compartments could be detected at 6 hpi. Therefore, 5dl1.2 can undergo viral DNA replication in MRC-5 cells and complementing cells, but replication cannot occur in Vero cells in the absence of ICP27.

After visualizing the differences in replication compartments in Vero and MRC-5 cells when ICP27 was absent, we more carefully quantified viral genome replication in MRC-5 and Vero cells, as well as N/TERT-2G, HFF, and HeLa cells. Viral genomes per cell were quantified as stated above (Fig. 1A). It is important to note that the black lines within these plots were shown earlier in Fig. 1A and are shown again for comparison (Fig. 4B through F). Consistent with imaging, minimal, if any, 5dl1.2 DNA replication occurred in Vero cells in the absence of ICP27 (Fig. 4B), but 5dl1.2 did undergo DNA replication in MRC-5 cells (Fig. 4C), albeit to a lesser extent than strain KOS. We also found that the timing and extent of DNA replication of 5dl1.2 varied depending on the cell type. In N/TERT-2G and HFF cells, infection with 5dl1.2 resulted in a slight defect in viral DNA replication (Fig. 4D and E). However, in HeLa cells, 5dl1.2 replication was minimal (Fig. 4F). Taken together, there is a cell type dependence on ICP27 for HSV-1 DNA replication.

### Viral gene expression defects during 5dl1.2 infection are consistent across cell types

**Fig 5:**
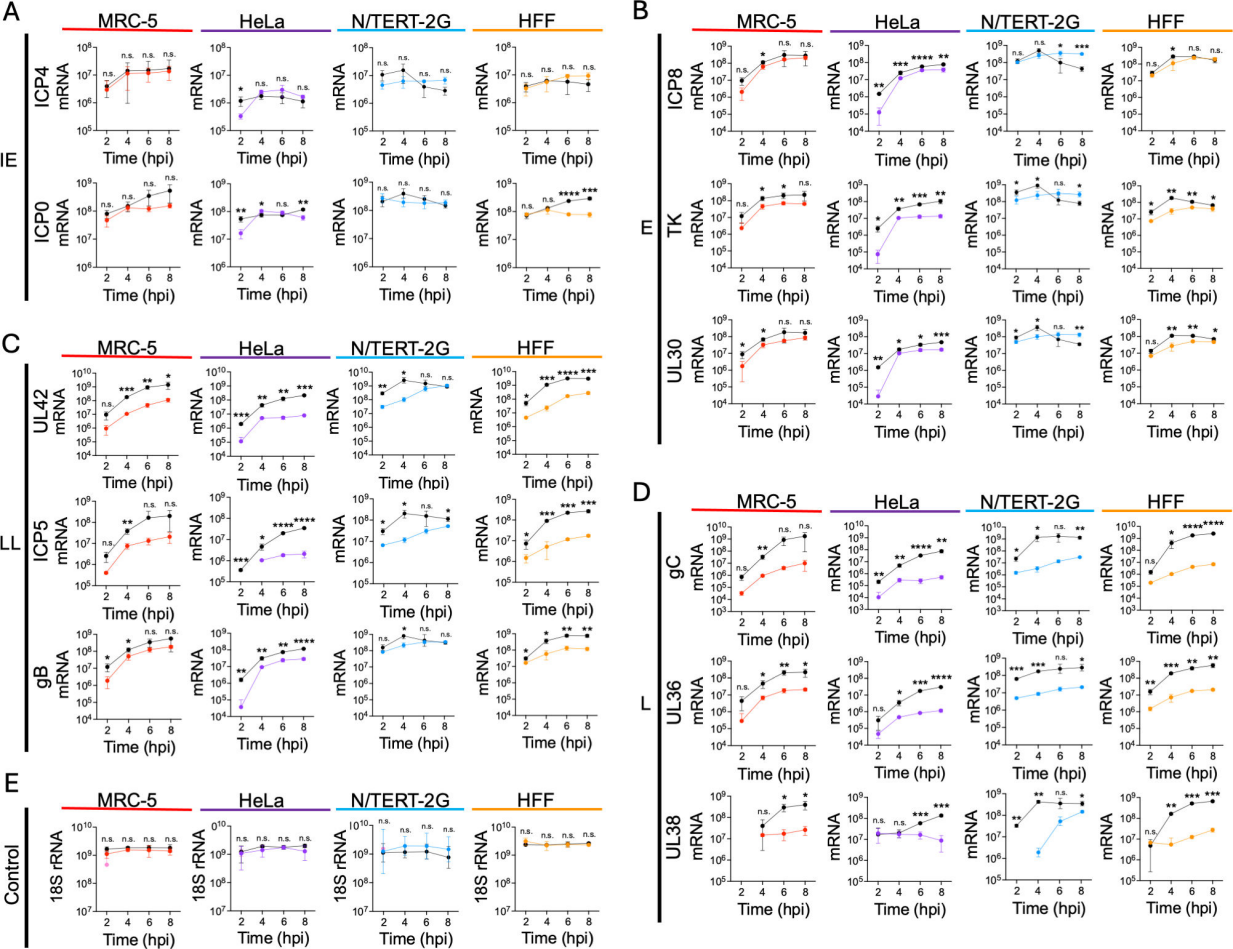
Viral gene expression kinetics during 5dl1.2 infection. MRC-5, HeLa, N/TERT-2G, or HFF cells were infected with 5dl1.2 or KOS at an MOI of 10 PFU/cell. Total RNA was isolated every 2 h for 8 h. After isolation, RNA was reverse-transcribed using oligo dT primers, and mRNAs were quantified by qPCR as follows: (**A**) IE genes, (**B**) E genes, (**C**) LL genes, and (**D**) L genes. (**E**) 18S rRNA was reverse-transcribed using a gene-specific primer and quantified by qPCR. Viral mRNA copies per microgram of total RNA were quantified relative to a standard curve for each gene. All values represent the mean with standard deviation (*n* = 3). Missing datapoints indicate that the mRNA was not detected above background levels. An unpaired two-tailed Student’s *t*-test was completed at each time point to compare mRNA copies per microgram of total RNA during KOS and 5dl1.2 infection (n.s. not significant, *P < 0.05, **P < 0.01, ***P < 0.001, ****P < 0.0001).

We next asked if there are cell type-specific differences in 5dl1.2 mRNA expression. We infected MRC-5, HeLa, N/TERT-2G, and HFF cells and quantified viral transcripts as described above (Fig. 5A through E, colored lines). The results from wild-type KOS infection are shown for comparison, and these data are the same as in Fig. 3 (black lines). If a point is not displayed on the graph, this indicates that the transcript was not detectable above background levels.

IE gene expression was not generally affected by the absence of ICP27, regardless of cell type, consistent with the literature (Fig. 5A). E gene expression was slightly reduced and was similar between cell types, where expression increased from 2 to 4 hpi and then plateaued from 4 to 8 hpi (Fig. 5B). Notably, the shutdown of E gene expression that was observed during KOS infection in N/TERT-2G cells did not occur during 5dl1.2 infection in these cells. As expected, LL and L mRNA expression was significantly reduced during infection with 5dl1.2 compared to KOS in all cell types tested (Fig. 5C and D). LL and L mRNA levels increased up to 8 hpi, but significantly fewer transcripts were produced in the absence of ICP27. LL mRNA expression in N/TERT-2G cells reached levels comparable to KOS infection by 8 hpi. Finally, there was a similar defect in L mRNA expression between the individual cell types. Taken together, gene expression defects during 5dl1.2 infection are shared by different cell types, including reduced E, LL, and L mRNA expression.

### Viral protein expression is altered across cell types during 5dl1.2 infection

**Fig 6:**
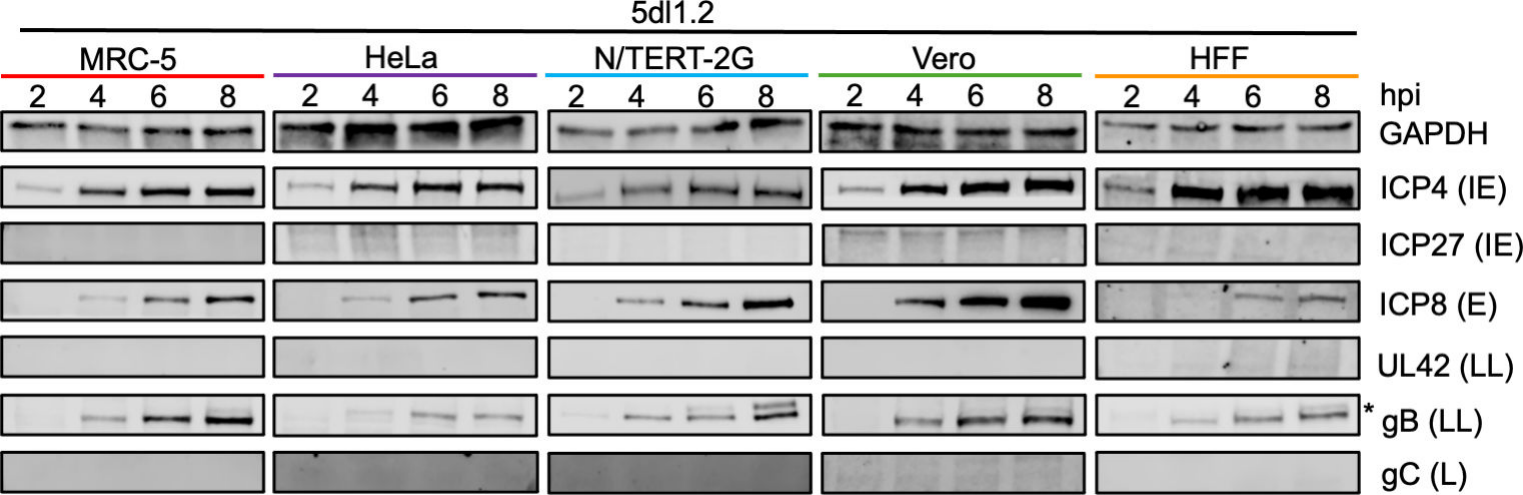
The absence of ICP27 results in undetectable UL42 and gC protein expression in each cell type. MRC-5, HeLa, N/TERT-2G, Vero, and HFF cells were infected with strain 5dl1.2 at an MOI of 10 PFU/cell. Total protein was collected at 2, 4, 6, or 8 hpi. Blots were incubated with antibodies indicated on the right of the images. * indicates a nonspecific band found in gB samples, the bottom band is the gB protein. 5dl1.2 samples were run, and blots probed together with KOS samples shown in Fig. 2.

We next examined the levels of viral protein expression during 5dl1.2 infection in each cell type (Fig. 6). Note that KOS and 5dl1.2 western blots were run, probed, and imaged together with the blots in Fig. 2 for each cell type. During 5dl1.2 infection, the expression of ICP4 is similar between cell types, and the timing of expression and levels are similar to KOS infection. In each cell type, ICP4 was detected at 2 hpi and leveled off around 4 hpi. This expression pattern is consistent with Fig. 5A, further showing IE protein expression is not impacted by the absence of ICP27. We also confirmed that 5dl1.2 does not produce detectable amounts of the ICP27 protein (Fig. 6). The representative E protein showed similar expression patterns across cell types. ICP8 was detected at 4 hpi and increased through 8 hpi in all cell types except HFF cells, where it was detected at 6 hpi. In N/TERT-2G, Vero, and HFF cells, ICP8 expression was similar regardless of KOS or 5dl1.2 infection, whereas 5dl1.2 infection in MRC-5 and HeLa cells resulted in ICP8 being expressed 2 h sooner than during KOS infection. We next examined LL protein expression during 5dl1.2 infection. UL42 mRNA was expressed at reduced levels compared to KOS (Fig. 5C), but no UL42 protein was detected during 5dl1.2 infection in all cell types tested (Fig. 6). On the other hand, although gB mRNA levels were reduced during 5dl1.2 infection (Fig. 5C), gB protein was still expressed close to KOS levels in all cell types (Fig. 6). Finally, although gC mRNA was expressed at low levels in all cell types tested during 5dl1.2 infection (Fig. 5D), the representative L protein was not detected during 5dl1.2 infection (Fig. 6). This is consistent with previous work that has shown that gC protein expression is dependent on ICP27 (33).

## DISCUSSION

In this study, we comprehensively investigated the nuclear stages of the HSV-1 infectious cycle in five cell types used for HSV-1 research (Vero, HeLa, HFF, MRC-5, and N/TERT-2G cells) and demonstrate how the infected cell type influences the outcome of infection. We have shown that HSV-1 viral DNA replication kinetics vary among cell types tested, although all cells have a similar maximum capacity to produce viral DNA (Fig. 1A). DNA replication in Vero, MRC-5, and HFF cells begins before 4 hpi and is mostly complete by 12 hpi. In HeLa cells, the number of infecting viral genomes decreases initially, and genome amplification is delayed. Although the number of viral genomes produced by 24 hpi is similar across cell types, HeLa cells produce less infectious virus, and HFF cells produce more infectious virus compared to other cell types (Fig. 1B and C). Also, HeLa cells express relatively less viral mRNA throughout infection (Fig. 3) and produce more defective virus particles by 24 hpi (Fig. 1D). In N/TERT-2G cells, viral DNA replication and gene expression begin earlier but plateau earlier as well. Together, these data demonstrate that while HSV-1 DNA replication capacity is conserved across cell types, cell type-specific differences in the kinetics of viral DNA replication, viral gene expression, and virion assembly contribute to the efficiency of infectious virus production.

In addition, we demonstrate that cell type can dramatically influence the infectious cycle of a viral mutant. Consistent with work from the Rice lab (18), we found that there are cell type-specific differences in the infectious cycle of an ICP27 mutant virus (5dl1.2). Although the defects in viral gene expression are similar between different cell types (Fig. 5 and 6), 5dl1.2 can replicate DNA to wild type levels in HFF cells, viral DNA replication is reduced in MRC-5 and N/TERT-2G cells, and little to no viral DNA replication can occur in HeLa and Vero cells (Fig. 4). Although the data are not shown, none of these cells can produce new infectious 5dl1.2 virions that are capable of infecting complementing or noncomplementing cells. Taken together, although ICP27 is required for viral gene expression across all cell types tested, DNA replication of an ICP27 null mutant occurs in a cell type-specific manner. These results underscore the importance of carefully selecting and characterizing the cell type used to study viral infection and viral mutants.

For this study, we selected a diverse panel of cell lines commonly used in HSV-1 research that differ in species origin, tissue type, and genetic characteristics. Vero cells originate from African green monkey tissue and were chosen as these cells are often used to prepare virus stocks and for plaque assays because they lack the ability to produce type I interferon (34–36). In contrast, HeLa, HFF, MRC-5, and N/TERT-2G cells were derived from human tissue. HeLa cells were the first immortalized cancer cell line and are used throughout HSV-1 research (16, 22, 23, 37–39). HFF and MRC-5 cells are primary fibroblast cells that are commonly used to study infection (13, 40–43) and N/TERT-2G cells are an immortalized keratinocyte cell line that could provide a reproducible and scalable model for HSV-1 research. Vero, HeLa, and N/TERT-2G cells are all epithelial, although derived from different tissues, including kidney, cervix, and skin, respectively. MRC-5 and HFF cells are fibroblasts derived from lung tissue and foreskin, respectively. Vero and HeLa cells are aneuploid (44, 45). Vero cells have fewer chromosomes than a typical cell, whereas HeLa cells have, on average, 78 chromosomes rather than 46 (45). Inversely, MRC-5, HFF, and N/TERT-2G cells are all diploid.

Vero, HeLa, and N/TERT-2G cells are all immortalized cell lines. Vero cells were immortalized by a spontaneous mutation due to continuous growth in cell culture (46). On the other hand, HeLa cells were immortalized due to the cancerous nature of the cells and contain an overactive telomerase enzyme, preventing the shortening of telomeres (39). HeLa cells have also integrated multiple incomplete copies of the human papillomavirus (HPV) genome that disrupt cellular mechanisms regulating lifespan and growth, contributing to cell immortality (47). N/TERT-2G cells were immortalized by introducing the human telomerase reverse transcriptase gene into primary human keratinocytes, accompanied by the spontaneous loss of the p16^INK4a^ cell cycle regulator (14, 48). This method of immortalizing these cells allows N/TERT-2G cells to remain diploid while dividing indefinitely (14). MRC-5 and HFF cells are not immortalized and have a finite life span in cell culture.

Another differentiating factor between the cell types used in this study is their interferon response. Vero cells cannot produce type I interferons (IFN-α and IFN-β) in response to viral infection, which makes these cells highly permissive to a wide range of viruses (34, 35). HeLa cells also have a compromised interferon response and generally produce lower levels of interferons compared to primary human cells (49). On the other hand, HFF, MRC-5, and N/TERT-2G cells appear to exhibit an intact IFN response (50–52).

In this study, all virus stocks were prepared in Vero or E11 cells, which are Vero cells that express ICP27 to complement the ICP27 mutation in 5dl1.2. However, an important consideration that was not explored in this study is how producer cell type influences infection outcome. This has recently been thoroughly investigated by the Taylor lab (53). In their study, they demonstrated that producer cell type can influence the capacity of a subsequently infected cell to produce viral transcripts, proteins, and infectious virions, and influences the protein composition of the virion and sensitivity to inhibition of replication by IFN treatment. After comparison of infection kinetics of virus produced in HFF, Vero, and HaCaT cells, they concluded that the optimal and most physiologically relevant producer cell type for HSV-1 is HaCaT cells, an immortalized keratinocyte cell line.

It is not our goal to indicate that one cell type is better than another to study HSV-1 infection, as all cell types grown in culture serve as models to understand the basic principles of infection. However, it is essential to understand the advantages and disadvantages of the model system, the infection kinetics for that model cell system, and the limitations and potential benefits of comparing data obtained from different cell types (54).

Here, we have shown that KOS and 5dl1.2 infectious cycles are influenced by the infected cell. Viral DNA replication is cell type-dependent during HSV-1 infection in the absence of ICP27. This suggests that cellular factors may contribute to viral DNA replication (40, 42, 55). Therefore, cellular proteins interacting with the viral replication fork could be different among cell types or other factors, such as stage of the cell cycle or protein modification state, may contribute. However, how this is influenced by cell type has not been carefully investigated. We have also shown that N/TERT-2G cells produce more viral transcripts than HFF, MRC-5, and HeLa cells during both strain KOS and 5dl1.2 infection. This may be due to more efficient transcription in N/TERT-2G cells and/or the earlier onset of viral DNA replication. N/TERT-2G cells are the most efficient at replicating viral DNA. We hypothesize that there are cellular factors present in keratinocytes that allow for this infectious cycle difference. Further work needs to be completed to understand the cellular factors present in each cell type that contribute to the efficiency of infection.

## MATERIALS AND METHODS

### Cells and viruses

HeLa, HFF, MRC-5, and Vero cells were obtained from and passaged as recommended by the American Type Culture Collection (ATCC). N/TERT-2G cells were obtained from Nir Drayman (University of California, Irvine). E11 cells, Vero cells that have been stably transfected with the HSV-1 ICP27 gene, were obtained from Neal DeLuca (University of Pittsburgh). HSV-1 strain KOS and strain 5dl1.2 were also obtained from Neal DeLuca (University of Pittsburgh) and were used to infect cells at an MOI of 10 PFU/cell unless otherwise stated. 5dl1.2 is a KOS derivative with a deletion that spans the BamHI site upstream of the ICP27 promoter until the first SalI site in the ICP27 gene (56). Strain KOS was propagated and titered in Vero cells, and strain 5dl1.2 was propagated and titered in E11 cells.

### DNA isolation and quantification

Cells were plated in a 12-well dish (2.5 × 10^5^ cells/well) and infected with either KOS or 5dl1.2 at an MOI of 10 PFU/cell. Every 2 h for 12 h and at 24 h, DNA was isolated using DNA extraction buffer (0.5% SDS, 400 μg/mL proteinase K, 100 mM NaCl). Real-time qPCR with SYBR green (Thermo Fisher, 4,367,659) was used to determine the number of viral genomes compared to a standard curve generated from purified viral DNA. Primers specific for the TK gene (Table 1) were used for viral DNA amplification. The number of cells present was determined relative to a standard curve generated from purified human DNA. Primers specific for the GAPDH gene (Table 1) were used for cellular DNA amplification. Note that HeLa cells have 4 copies of GAPDH per cell, while diploid cells have 2 (57).

**TABLE 1:** Gene specific primers

Gene	Forward primer (5´−3´)	Reverse primer (5´−3´)
ICP4	CCACGGGCCGCTTCA	GCGATAGCGCGCGTAGAA
ICP27	GTCTCCTGGGAAACCTTGGTC	GAAATTTTCTTGGCGCAGCAC
ICP0	GTCGCCTTACGTGAACAAGAC	GTCGCCATGTTTCCCGTCTG
ICP8	CATCAGCTGCTCCACCTCGCG	GCAGTACGTGGACCAGGCGGT
TK	CCAAAGAGGTGCGGGAGTTT	ACCCGCTTAACAGCGTCAACA
UL30	CATCACCGACCCGGAGAGGGAC	GGGCCAGGCGCTTGTTGGTGT
UL42	ACGTCCGACGGCGAGG	CAGGCGCAACTGAACGTC
ICP5	TGGATGGTATGGTCCAGATGC	GCACAACGGCGCTGCTCT
gB	TACTGCTGGCCCACCTTG	GCTCTCGCGCGTGGACCTG
gC	GTGACGTTTGCCTGGTTCCTGG	GCACGACTCCTGGGCCGTAACG
GAPDH	CAGAACATCATCCCTGCCTCTACT	GCCAGTGAGCTTCCCGTTCA
18S rRNA	TACCACATCCAAGGAAGGCAGCA	TGGAATTACCGCGGCTGCTGGCA

### Analysis of viral yield

For high MOI infection, cells were plated at 2.5 × 10^5^ cells/well in a 12-well dish and infected with strain KOS at an MOI of 10 PFU/cell. At 24 hpi, cells were collected by scraping and freeze-thawed three times and then sonicated to release cell-associated virus. Viral yield was determined by plaque assay in Vero cells.

For low MOI infection, cells were plated at 2.5 × 10^5^ cells/well in a 12-well dish and infected with strain KOS at an MOI of 0.01 PFU/cell. At 24, 48, 72, and 96 hpi, cells were collected by scraping and freeze-thawed three times, followed by sonication to release cell-associated virus. Viral yield was determined by plaque assay in Vero cells.

### Genome to PFU quantification

Cells were plated at 2.5 × 10^5^ cells/well in a 12-well dish and infected with strain KOS at an MOI of 10 PFU/cell. At 24 hpi, cells were collected by scraping, centrifuged, and the supernatant was transferred to a new tube. An aliquot of supernatant was used to determine viral yield by plaque assay in Vero cells. The remainder of the supernatant was subject to a DNase I (Sigma, 11284932001) treatment to ensure the quantification of DNA only includes encapsidated DNA. Following heat inactivation of DNase I, the viral capsids were lysed using a proteinase K solution (0.5% SDS, 20 mg/mL proteinase K). Proteinase K was heat-inactivated, and the samples were diluted and subject to qPCR using SYBR green (Thermo Fisher, 4367659) to determine the number of viral genomes present compared to a standard curve generated from purified viral DNA. Primers specific for the TK gene (Table 1) were used for viral DNA amplification.

### Protein isolation and western blotting

Cells were plated at 2.5 × 10^5^ cells/well in 12-well dishes and were infected with either 5dl1.2 or KOS at an MOI of 10 PFU/cell. Protein was isolated at 2, 4, 6, and 8 hpi using Laemmli SDS sample buffer. Western blotting was carried out using the following primary antibodies: anti-ICP4 (Abcam ab6514, 1:1,000), anti-ICP27 (Virusys P1113, 1:100), anti-ICP8 (Abcam ab20194, 1:1,000), anti-UL42 (Abcam ab19311, 1:500), anti-gB (Abcam ab6506, 1:1,000), anti-gC (Fisher 01-676-023, 1:500), and anti-GAPDH (Invitrogen AM4300, 1:5,000).

### RNA isolation and quantification

Cells were plated at 1 × 10^6^ cells/well in six-well dishes. Cells were infected with either strain KOS or 5dl1.2 at an MOI of 10 PFU/mL, and total RNA was isolated at indicated times using TRIzol according to the manufacturer’s protocol (58). Following solubilization, RNA was treated with the DNA-free DNA Removal Kit (Invitrogen, AM1906). RNA quantity was determined by Qubit High Sensitivity RNA assay (58). The quality of the RNA was verified by Tapestation (Agilent). 500 ng of RNA was reverse-transcribed with an oligo dT primer (58) or a gene-specific 18S reverse transcription primer (18S rRNA RT 5’–GAGCTGGAATTACCGCGGCTGCTGGCA - 3’) (59) using M-MLV reverse transcriptase (Invitrogen, 28025013). The number of viral transcripts present was determined by qPCR using SYBR green (Thermo Fisher, 4367659) in comparison to a standard curve generated from purified viral DNA. See Table 1 for gene-specific primers (60).

### Immunofluorescence imaging

Imaging was carried out as previously described (40). E11, MRC-5, and Vero cells were plated at a low density (1.67 × 10^5^) on coverslips. Cells were infected with 5dl1.2 containing EdC-labeled viral DNA at an MOI of 10 PFU/cell. Cells were fixed with 4% paraformaldehyde at 6 hpi. Cellular chromatin was stained with Hoechst, viral DNA binding protein ICP4 was labeled by indirect immunofluorescence, and infecting viral DNA was visualized by click chemistry. Images were acquired on a Nikon Eclipse Ti2 inverted confocal microscope.

## Data Availability

All data are included in the article.

## SUPPLEMENTAL MATERIAL

Figure S1 jvi.00052-26-s0001.pdf Statistical analysis of the comparative expression of IE and E viral genes.

Figure S2 jvi.00052-26-s0002.pdf Statistical analysis of the comparative expression of LL and L viral genes and control RNAs.

ASM does not own the copyrights to Supplemental Material that may be linked to, or accessed through, an article. The authors have granted ASM a non-exclusive, world-wide license to publish the Supplemental Material files. Please contact the corresponding author directly for reuse.
