# Supplementary material for: Cell type-specific differences in herpes simplex virus type 1 infection and dependency on ICP27
Source: J Virol. 2026 Feb 26;100(3):e00052-26. doi: 10.1128/jvi.00052-26 (PMC13011426; doi:10.1128/jvi.00052-26)
Supplement: Figure S1 — Statistical analysis of the comparative expression of IE and E viral genes. [file jvi.00052-26-s0001.pdf]

A

IE

ICP27

Cell type 1	Cell type 2	Time (hpi)			
		2	4	6	8
MRC-5	HeLa	ns	ns	***	*
MRC-5	N/TERT-2G	**	ns	***	*
MRC-5	HFF	ns	ns	***	*
HeLa	N/TERT-2G	***	*	ns	ns
HeLa	HFF	ns	ns	ns	ns
N/TERT-2G	HFF	**	ns	ns	ns

ICP4

Cell type 1	Cell type 2	Time (hpi)			
		2	4	6	8
MRC-5	HeLa	ns	ns	ns	ns
MRC-5	N/TERT-2G	ns	ns	ns	ns
MRC-5	HFF	ns	ns	ns	ns
HeLa	N/TERT-2G	**	ns	ns	ns
HeLa	HFF	ns	ns	ns	ns
N/TERT-2G	HFF	ns	ns	ns	ns

ICP0

Cell type 1	Cell type 2	Time (hpi)			
		2	4	6	8
MRC-5	HeLa	ns	ns	ns	ns
MRC-5	N/TERT-2G	*	*	ns	ns
MRC-5	HFF	ns	ns	ns	ns
HeLa	N/TERT-2G	*	**	ns	ns
HeLa	HFF	ns	ns	ns	ns
N/TERT-2G	HFF	*	*	ns	ns

B

E

ICP8

Cell type 1	Cell type 2	Time (hpi)			
		2	4	6	8
MRC-5	HeLa	ns	ns	ns	ns
MRC-5	N/TERT-2G	***	**	ns	ns
MRC-5	HFF	ns	ns	ns	ns
HeLa	N/TERT-2G	***	***	ns	ns
HeLa	HFF	ns	ns	ns	ns
N/TERT-2G	HFF	**	*	ns	ns

TK

Cell type 1	Cell type 2	Time (hpi)			
		2	4	6	8
MRC-5	HeLa	ns	ns	ns	ns
MRC-5	N/TERT-2G	***	**	ns	ns
MRC-5	HFF	ns	ns	ns	ns
HeLa	N/TERT-2G	***	**	ns	ns
HeLa	HFF	ns	ns	ns	ns
N/TERT-2G	HFF	***	**	ns	ns

UL30

Cell type 1	Cell type 2	Time (hpi)			
		2	4	6	8
MRC-5	HeLa	ns	ns	ns	ns
MRC-5	N/TERT-2G	****	**	ns	ns
MRC-5	HFF	ns	ns	ns	ns
HeLa	N/TERT-2G	****	**	ns	ns
HeLa	HFF	ns	ns	ns	ns
N/TERT-2G	HFF	***	**	ns	ns

Supplementary Figure 1. Statistical analysis of the comparative expression of IE and E viral genes. A one-way ANOVA with Tukey's multiple comparisons test was completed to compare the differences in mRNA expression between each cell type presented in the graphs in Figure 3 (ns not significant, * $p<0.0.05$, ** $p <0.01$, *** $p<0.001$, **** $p<0.0001$). A) IE genes B) E genes.
