# Supplementary material for: Cell type-specific differences in herpes simplex virus type 1 infection and dependency on ICP27
Source: J Virol. 2026 Feb 26;100(3):e00052-26. doi: 10.1128/jvi.00052-26 (PMC13011426; doi:10.1128/jvi.00052-26)
Supplement: Figure S2 — Statistical analysis of the comparative expression of LL and L viral genes and control RNAs. [file jvi.00052-26-s0002.pdf]

A

LL

UL42

Cell type 1	Cell type 2	Time (hpi)			
		2	4	6	8
MRC-5	HeLa	ns	ns	ns	*
MRC-5	N/TERT-2G	****	**	ns	ns
MRC-5	HFF	ns	ns	***	**
HeLa	N/TERT-2G	****	**	*	ns
HeLa	HFF	ns	ns	****	***
N/TERT-2G	HFF	***	ns	**	***

ICP5

Cell type 1	Cell type 2	Time (hpi)			
		2	4	6	8
MRC-5	HeLa	ns	ns	ns	ns
MRC-5	N/TERT-2G	***	**	ns	ns
MRC-5	HFF	ns	ns	ns	ns
HeLa	N/TERT-2G	***	**	ns	ns
HeLa	HFF	ns	ns	ns	*
N/TERT-2G	HFF	**	ns	ns	ns

gB

Cell type 1	Cell type 2	Time (hpi)			
		2	4	6	8
MRC-5	HeLa	ns	ns	ns	ns
MRC-5	N/TERT-2G	**	**	ns	ns
MRC-5	HFF	ns	ns	ns	ns
HeLa	N/TERT-2G	***	**	ns	ns
HeLa	HFF	ns	ns	*	ns
N/TERT-2G	HFF	**	ns	ns	ns

B

L

gC

Cell type 1	Cell type 2	Time (hpi)			
		2	4	6	8
MRC-5	HeLa	ns	ns	ns	ns
MRC-5	N/TERT-2G	**	*	ns	ns
MRC-5	HFF	ns	ns	ns	ns
HeLa	N/TERT-2G	**	*	ns	ns
HeLa	HFF	ns	ns	*	*
N/TERT-2G	HFF	**	ns	ns	ns

UL36

Cell type 1	Cell type 2	Time (hpi)			
		2	4	6	8
MRC-5	HeLa	ns	ns	ns	ns
MRC-5	N/TERT-2G	****	***	ns	ns
MRC-5	HFF	ns	***	ns	*
HeLa	N/TERT-2G	****	****	ns	ns
HeLa	HFF	*	****	*	**
N/TERT-2G	HFF	****	ns	ns	ns

UL38

Cell type 1	Cell type 2	Time (hpi)			
		2	4	6	8
MRC-5	HeLa	ns	ns	ns	ns
MRC-5	N/TERT-2G	*	****	ns	ns
MRC-5	HFF	ns	*	ns	*
HeLa	N/TERT-2G	ns	****	ns	ns
HeLa	HFF	ns	**	*	***
N/TERT-2G	HFF	*	***	ns	*

C

Control

18S

Cell type 1	Cell type 2	Time (hpi)			
		2	4	6	8
MRC-5	HeLa	ns	ns	ns	ns
MRC-5	N/TERT-2G	ns	ns	ns	ns
MRC-5	HFF	ns	ns	ns	ns
HeLa	N/TERT-2G	ns	ns	ns	ns
HeLa	HFF	ns	ns	ns	ns
N/TERT-2G	HFF	ns	*	ns	**

GAPDH

Cell type 1	Cell type 2	Time (hpi)			
		2	4	6	8
MRC-5	HeLa	ns	ns	ns	ns
MRC-5	N/TERT-2G	**	**	*	**
MRC-5	HFF	ns	ns	ns	*
HeLa	N/TERT-2G	*	**	ns	**
HeLa	HFF	ns	ns	ns	*
N/TERT-2G	HFF	ns	*	ns	ns

Supplementary Figure 2. Statistical analysis of the comparative expression of LL and L viral genes and control RNAs. A one-way ANOVA with Tukey's multiple comparisons test was completed to compare the differences in mRNA expression between each cell type presented in the graphs in Figure 3 (ns not significant, * p<0.0.05, ** p <0.01, *** p<0.001, **** p<0.0001). A) LL genes B) L genes C) controls.
